# Quality of life in children undergoing tonsillectomy: a cross-sectional survey

**DOI:** 10.1186/s13052-023-01449-0

**Published:** 2023-05-04

**Authors:** Ying Zhou, Mingqi Peng, Jing Zhou

**Affiliations:** Department of Nursing, Children’s Hospital of Nanjing Medical University, No. 72, Guangzhou Road, Gulou District, Nanjing, Jiangsu Province China

**Keywords:** Quality of life, Children, Tonsillectomy, Care, Nursing, Clinical

## Abstract

**Background:**

The quality of life in children undergoing tonsillectomy remains unclear. We aimed to analyze the current status and influencing factors of quality of life in children undergoing tonsillectomy, to provide useful insights to clinical postoperative care for children.

**Methods:**

Children who underwent tonsillectomy in our hospital from June 1, 2021 to October 31, 2022 were selected. The characteristics of children were collected and Paediatric Quality of Life Inventory Version 4.0 (PedsQL™ 4.0) was used for evaluating the quality of life in children. Pearson correlation, univariate and logistic regression analyses were condcuted to evaluate the influencing factors of quality of life in children undergoing tonsillectomy.

**Results:**

A total of 118 children undergoing tonsillectomy were included. The total score of PedsQL™ 4.0 in children undergoing tonsillectomy was (70.28 ± 13.15). Pearson correlation analyses indicated that age(r = 0.586), courses of tonsillitis(r = 0.595), parental education level(r = 0.612) and monthly family income(r = 0.608) were associated with the PedsQL™ 4.0 total score in children undergoing tonsillectomy (all P < 0.05). Logistic regression analyses indicated that age ≤ 5y (OR = 2.009,95%CI: 1.826 ~ 2.401), courses of tonsillitis ≥ 3years (OR = 2.167,95%CI: 1.688 ~ 2.547), high school of parental education level (OR = 1.807,95%CI: 1.224 ~ 2.181) and monthly family income ≤ 5000 RMB(OR = 2.624,95%CI:2.092 ~ 3.077) were the independent influencing factors of quality of life in children with undergoing tonsillectomy (all P < 0.05).

**Conclusions:**

The quality of life of children after tonsillectomy is not high, and the quality of life of children is affected by many factors. Medical staff should take early targeted nursing countermeasures tageted on those influencing factors to improve the quality of life of children.

**Supplementary Information:**

The online version contains supplementary material available at 10.1186/s13052-023-01449-0.

## Background

In recent years, the incidence of tonsillitis and enlarged tonsil is increasing with the aggravation of environmental pollution [1]. Besides, passive smoking [2] and infectious agents use [3] may be closely associated with the development of tonsillitis. Chronic tonsillitis is a long term condition that consists of certain symptoms, including generalized swelling and inflammation of the pharyngeal tonsils and the back of the throat [4]. As a common clinical disease in otorhinolaryngology, children with chronic tonsillitis can show long-term pharyngeal discomfort and foreign body sensation, dry and itchy throat, irritant cough, halitosis and other clinical symptoms, which seriously affect their health and quality of life [5, 6]. In severe cases, it can even be complicated with heart and kidney diseases, threatening the life safety of patients [7]. Therefore, the timely and effective treatment are essential to the prognosis of children with tonsillitis [8].

Tonsillectomy is a common treatment for chronic tonsillitis at present, and the quality of life of postoperative patients is one of the key points of clinical concern [9, 10]. At present, there are few reports on the predictors of quality of life in patients with chronic tonsillitis after tonsillectomy at home and abroad. The quality of life of patients with tonsillitis after tonsillectomy remains unclear. The analysis of influencing factors of quality of life after tonsillectomy in children with chronic tonsillitis can provide a basis for nursing care and improvement of quality of life of patients with chronic tonsillitis after tonsillectomy. Therefore, this study aimed to analyze the factors affecting the quality of life of children with tonsillitis after tonsillectomy, to provide guidance and evidence support for nursing care to improve the quality of life of children undergoing tonsillectomy.

## Methods

This study was a a cross-sectional survey design. This study was conducted and reported in compliance with the related regulations and guidelines.The study protocol had been verified and approved by the ethic committee of our hospital on May 15, 2021 with approval number: 201601003-1.1. In this study, the written informed consents were obtained from all the included children and their guardians. The data collected in this study are only used for the analysis of this study and would not divulge the relevant personal information of the children for any purpose.

### Patients

In this study, children who underwent tonsillectomy in our hospital from June 1, 2021 to October 31, 2022 were selected as the study population. The inclusion criteria of the children were as follows: the child met the standard of tonsillectomy or chronic tonsillectomy in the American 2011 Clinical practice Guide for Children, and underwent surgery treatment in our hospital; the age of the child was 2 to 13 years old; the child and his family members knowingly and agreed to participate in this study. The exclusion criteria of this study were as follows: children with nasopharyngeal carcinoma, nasopharyngeal fibroangioma and other nasopharyngeal diseases; children with primary systematic diseases associated with heart, liver, kidney and brain; And children who did not agree to participate in this study were excluded in this study.

### Tonsillectomy and care

The children were taken supine position and the operation was carried out under oral intubation and intravenous anesthesia. All patients were treated with hypothermic plasma operation system, cutting power was 6 grades, blood coagulation power was 6 grades, and routine disinfection and toweling were performed after satisfactory anesthesia. The stretcher opened the mouth and fully exposed the bilateral tonsils. The plasma knife cut the mucosa along the mucosal junction of the palatolingual arch and the tonsil, exposed the tonsillar capsule, and then removed the tonsil completely along the capsule. During the operation, the bleeding point was completely stopped by using a coagulation stall. For children with acute pain, we use 0.3 g ibuprofen (Sino-American Tianjin SmithKline Pharmaceuticals, Tianjin, China) once every 4 to 6 h, with a maximum of 2.4 g / day. All children were given thrombin and dexamethasone 2 days after operation, budesonide suspension was atomized, cold liquid food was ordered 6 h after operation, semi-fluid diet was allowed on the 2nd day, the temperature should not be too high, and soft food was taken from the 3rd day to the period from the 3rd day to the complete exfoliation of the albino.

### Survey

We used following tools to take survey to children one month after tonsillectomy when the child comes back for re-check.

General data questionnaire: It was designed and completed by the staff of the research group according to the results of literature review and completed by the guardian of the child. It mainly included the following contents: Age, gender, body mass index (BMI), whether the child was the only child of family, place of residence, guardians, courses of tonsillitis, parental education level and monthly family income.

Paediatric Quality of Life Inventory Version 4.0 (PedsQL™ 4.0) [11]: The scale has 4 dimensions and 23 items, including physiological function (8 items), emotional function (5 items), social function (5 items) and school function (5 items). Each item of the scale is a survey of the frequency of an event in the last month. The answer options have 5 grades, and the score is correspondingly converted to 100: 0. The score of each dimension is the sum of the scores of the answered items divided by the number of items answered. The total score and the score of each dimension is 0,100. The higher the score, the better the quality of life [12]. Chinese scholar Lu et al [13]. have modified and verified the Chinese version of the self-rating scale PedsQL™ 4.0. The Cronbach’s α coefficient of PedsQL™ 4.0 is between 0.74 and 0.82. The scale has good discriminant validity and construct validity.

We provided unified training to the investigators, properly organized, explained, guided and checked the questionnaire collection, and input the collected data after being checked by two researchers. Before the distribution of the questionnaire, the investigators introduced the purpose and significance of the study to the children and their parents, and obtained the consent of the parents and the children were willing to participate in the survey.

### Statistical analysis

The data of this study were statistically processed by SPASS23.0 software. The measurement data were expressed by mean ± standard deviation, and t-test was used for comparison between groups. The counting data were expressed as percentage and were analyzed by chi-square or rank sum test. Pearson correlation analyses were conducted to evaluate the correction of of PedsQL™ 4.0 total score and characteristics of children undergoing tonsillectomy. Univariate analysis and logistic regression analyses were performed to evaluate the influencing factors of quality of life in children with undergoing tonsillectomy. In this study, P < 0.05 was taken as the significant difference between groups.

## Results

### The characteristics of included children

A total of 118 children undergoing tonsillectomy were included. The average age of the children was 6.02 ± 2.43 years old, the average courses of tonsillitis was 2.42 ± 1.27 years.The characteristics of children undergoing tonsillectomy are presented in Table 1.

**Table 1:** The characteristics of children undergoing tonsillectomy(n = 118)

Variables	Characteristics
Age(y)	6.02 ± 2.43
Male/female	71/47
BMI (kg/m^2^)	21.15 ± 2.54
Only child of family	
Yes	55(46.61%)
No	63(53.39%)
Place of residence	
Rural area	68(57.63%)
City	50(42.37%)
Guardians	
Parents	72(61.02%)
Grandparents	39(33.05%)
Others	7(5.93%)
Courses of tonsillitis(years)	2.42 ± 1.27
Parental education level	
High school	33(27.97%)
Junior college	58(49.15%)
Undergraduate	20(16.95%)
Master or doctor	7(5.93%)
Monthly family income (RMB)	
≤ 5000	46(38.98%)
> 5000	72(61.02%)

BMI, body mass index

### PedsQL™ 4.0 score

The total score of PedsQL™ 4.0 score in children undergoing tonsillectomy was (70.28 ± 13.15). The score of school function dimension is the lowest and the social function dimension is the highest in PedsQL™ 4.0 score. The score details are shown in Table 2.

**Table 2:** The PedsQL™ 4.0 score in children undergoing tonsillectomy

Items	Score
Physiological function	64.36 ± 18.21
Emotional function	76.11 ± 19.52
Social function	81.08 ± 15.77
School function	61.35 ± 18.62
Total average score	70.28 ± 13.15

As indicated in Table 3, there were statistical differences in the PedsQL™ 4.0 total score between children with different age, courses of tonsillitis, parental education level and monthly family income (all P < 0.05). No significant differences in the PedsQL™ 4.0 total score between children with different gender, BMI, only child of family, residence place were found (all P > 0.05).

**Table 3:** Univariate analysis on the PedsQL™ 4.0 total score and characteristics of children undergoing tonsillectomy

Variables	PedsQL™ 4.0 total score	t/F	P
Age(y)		12.053	0.007
≤ 5	62.05 ± 12.11		
> 5	75.29 ± 16.09		
Gender		11.295	0.097
Male	71.18 ± 16.26		
Female	68.95 ± 14.72		
BMI (kg/m^2^)		10.055	0.128
<20	69.15 ± 13.48		
≥ 20	70.88 ± 14.13		
Only child of family		12.272	0.114
Yes	71.04 ± 14.82		
No	68.46 ± 12.55		
Place of residence		11.417	0.086
Rural area	70.12 ± 12.27		
City	71.04 ± 14.82		
Guardians		13.295	0.103
Parents	70.49 ± 11.67		
Grandparents	71.22 ± 12.96		
Others	69.05 ± 13.71		
Courses of tonsillitis (years)		12.755	0.031
≥ 3	67.69 ± 12.94		
< 3	71.81 ± 13.25		
Parental education level		10.285	0.026
High school	67.23 ± 12.78		
Junior college	70.11 ± 10.26		
Undergraduate	71.07 ± 12.82		
Master or doctor	71.11 ± 13.04		
Monthly family income (RMB)	70.12 ± 12.27	14.006	0.019
≤ 5000	68.06 ± 13.18		
> 5000	71.29 ± 10.79		

BMI, body mass index

### Influencing factors of quality of life in children with undergoing tonsillectomy

As showed in Table 4, Pearson correlation analyses indicated that age(r = 0.586), courses of tonsillitis(r = 0.595), parental education level(r = 0.612) and monthly family income(r = 0.608) were associated with the PedsQL™ 4.0 total score in children undergoing tonsillectomy (all P < 0.05).

**Table 4:** Pearson correlation analysis of PedsQL™ 4.0 total score and characteristics of children undergoing tonsillectomy

Variables	r	P
Age(y)	0.586	0.031
Gender	0.101	0.102
BMI (kg/m^2^)	0.116	0.131
Only child of family	0.124	0.087
Place of residence	0.209	0.154
Guardians	0.163	0.098
Courses of tonsillitis (years)	0.595	0.037
Parental education level	0.612	0.014
Monthly family income (RMB)	0.608	0.022

BMI, body mass index

As showed in Table 5, logistic regression analyses indicated that age ≤ 5y(OR = 2.009,95%CI: 1.826 ~ 2.401 ), courses of tonsillitis ≥ 3years(OR = 2.167,95%CI: 1.688 ~ 2.547), high school of parental education level(OR = 1.807,95%CI: 1.224 ~ 2.181) and monthly family income ≤ 5000 RMB(OR = 2.624,95%CI:2.092 ~ 3.077) were the independent influencing factors of quality of life in children with undergoing tonsillectomy (all P < 0.05).

**Table 5:** Logistic regression analysis on the influencing factors of quality of life in children with undergoing tonsillectomy

Variables	β	Wald	OR	95%CI	P
Age ≤ 5y	0.116	0.258	2.009	1.826 ~ 2.401	0.012
Courses of tonsillitis ≥ 3years	0.109	0.189	2.167	1.688 ~ 2.547	0.019
High school of parental education level	0.121	0.133	1.807	1.224 ~ 2.181	0.026
Monthly family income ≤ 5000 RMB	0.113	0.195	2.624	2.092 ~ 3.077	0.032

## Discussions

Tonsillectomy is one of the most common surgeries in children, with about 500,000 children undergoing tonsillectomy each year because of recurrent tonsillar infections or snoring in children [14]. Tonsillectomy can improve the symptoms of chronic tonsillitis and snoring in children with enlarged tonsil, but postoperative complications such as fever, pain, reduced diet and activity, and bleeding cannot be ignored [15–17]. Ibuprofen resulted to be the most studied nonsteroidal anti-inflammatory drug in the management of acute pain in children. It has been reported that the role of ibuprofen in the management of postoperative pain and, particularly, after tonsillectomy and/or adenoidectomy has been reconsidered recently [18]. Therefore, the quality of life and nursing care of children in children with undergoing tonsillectomy requires further investigations.

Combined with the standardized scale, this study has analyzed the quality of life of children undergoing tonsillectomy according to their individual and family social demographic characteristics, and discussed the factors affecting the quality of life of children undergoing tonsillectomy. It has a certain guiding significance for the clinical comprehensive intervention of tic disorder. The PedsQL used in this study is a systematic measurement tool to study children’s quality of life, which is widely used [19]. The general applicable core scale for measuring the common part of children’s quality of life has been proved to be suitable for measuring the quality of life of children suffering from some acute and chronic diseases, and has good reliability and validity [20].The results of this study have that the quality of life in children undergoing tonsillectomy is not high. Besides, age, courses of tonsillitis, parental education level and monthly family income are associated with the quality of life in children undergoing tonsillectomy. For children with age ≤ 5y, courses of tonsillitis ≥ 3years, high school of parental education level and monthly family income ≤ 5000 RMB, they may have lower quality of life in children with undergoing tonsillectomy. Medical staff should make targeted evaluation and intervention on the influencing factors, timely take effective nursing care to improve their quality of life.

In this study, the age and duration of illness had a significant impact on the quality of life. Studies [21, 22] on the quality of life reported by children aged 2–18 or 5–18 and their parents have shown that age is one of the factors affecting children’s quality of life, and children aged 2 to 5 will affect their normal development because of serious illness, invasive operation and academic interruption. It has been found that there are great changes in disease-specific and global quality of life for children with recurrent or chronic tonsillitis at 6 months and 1 year after tonsillectomy [23, 24]. The inconsistency of age research results may be related to different age groups, different research tools or different family structure. The longer the courses of tonsillitis, and the mucosal lesions caused by long-term inflammatory stimulation are relatively severe [25], so the quality of life of children within one month is relatively poor, and the results of follow-up may be improved. Therefore, in clinical care, for children with younger age and longer course of disease, the nurses should follow up the changes of children’s condition as soon as possible, strengthen the education of disease-related knowledge and pay attention to the quality of life of children [26–28].

The results of this study show that the educational level of parents and family income are the factors that affect the quality of children undergoing tonsillectomy. The higher the average monthly income of the family, the higher the quality of life of the children, which may be due to the higher the educational level of the parents, the better the family’s economy, the higher and more effective treatment and timely and effective medical resources, which improve the quality of life of the children [29, 30]. The education level of the guardian of the child is the factor affecting the quality of life of the child, which is different from the findings of other studies [31, 32]. As the child’s educational level and cognitive ability are not perfect, the guardian’s cognition of the disease, psychological state and handling style in the process of coping will exert an imperceptible influence on the child [33–35]. Some studies [36–38] have shown that the psychological reaction of parents is an important factor affecting children’s behavior, and the education level of guardians is an important factor affecting their cognition and coping. In this study, parents account for the highest proportion of guardians in this study. Parents of children with chronic tonsillitis bear multiple pressures such as hope, stress, and children’s studies in the long process of treatment. Parents’ poor coping and emotional problems will have adverse effects on children’s somatic diseases [39, 40]. The main caregivers with different levels of education have different degrees of understanding of the illness of the children, which leads to different flexibility in dealing with family emergencies [41]. Therefore, medical staff should guide and coordinate the relevant factors that affect the family resilience of children, and give correct and targeted support means and resources to children’s families and members, so as to reduce the impact of disease on children’s families, and improve the treatment effect and healthy outcome of children from the family point of view [42, 43]. Besides, clinical staff should understand the views and coping styles of guardians with different levels of education on the disease, especially those with low level of education or poor coping, so as to avoid the influence of guardians’ negative ideas and emotions on children as far as possible, to create a relatively warm and positive environment for children [44–46].

There are some limitations in this study that are worth considering. First of all, the samples of this study come from a single center, and the sample size is small, so we need to further explore and analyze the factors affecting the quality of life of children after tonsillectomy in the future. Secondly, the relevant factors included in this study are relatively limited, and there may be other factors such as exposure to passive smoke, other environmental factors, or allergy, which may affect the prognosis of children undergoing tonsillectomy, which need to be further studied in the future. Finally, this study is a cross-sectional survey, cross-sectional assessment of the current situation cannot determine causality, and further prospective studies are needed to verify the intervention effects of these influencing factors.

## Conclusions

In summary, we have found that the quality of life in children undergoing tonsillectomy is not satisfactory. Age ≤ 5y, courses of tonsillitis ≥ 3years, lower parental education level and monthly family income ≤ 5000 RMB are the influencing factors of quality of life in children undergoing tonsillectomy. In view of those influencing factors, clinical medical staff should take early, timely and effective interventions and nursing measures to improve the quality of life of children. In the future, related studies still need to further expand the sample size, improve the case data of children, and prolong the follow-up time, so as to draw more accurate conclusions and provide evidence support for clinical postoperative nursing.

## Electronic supplementary material

Below is the link to the electronic supplementary material.

Supplementary Material 1

Supplementary Material 2

## Data Availability

All data generated or analyzed during this study are included in this published article.

## List of abbreviations

PedsQL™ 4.0: Paediatric Quality of Life Inventory Version 4.0
BMI: Body mass index

## References

[1] Hulse K, Lindsay E, Rogers A, Young D, Kunanandam T, Douglas CM (2022). Twenty-year observational study of paediatric tonsillitis and tonsillectomy. Arch Dis Child.

[2] Marseglia GL, Avanzini MA, Caimmi S, Caimmi D, Marseglia A, Valsecchi C, Poddighe D, Ciprandi G, Pagella F, Klersy C (2009). Passive exposure to smoke results in defective interferon-gamma production by adenoids in children with recurrent respiratory infections. J Interferon Cytokine Res.

[3] Zuo L, He L, Huang A, Liu Y, Zhang A, Wang L, Song Y, Geng J (2022). Risk factors and antibiotic sensitivity of aerobic bacteria in chinese children with adenoid hypertrophy. BMC Pediatr.

[4] Palchun VT, Kryukov AI, Gurov AV, Kelemetov AA, Ermolaev AG, Muratov DL (2022). [Modern approaches to the surgical treatment of chronic tonsillitis]. Vestn Otorinolaringol.

[5] Ferguson M, Aydin M, Mickel J (2014). Halitosis and the tonsils: a review of management. Otolaryngol Head Neck Surg.

[6] Foki E, Seemann R, Stelter K, Lill C (2017). The effect of tonsillotomy on chronic recurrent tonsillitis in children. Acta Otolaryngol.

[7] Norton L, Myers A (2021). The treatment of streptococcal tonsillitis/pharyngitis in young children. World J Otorhinolaryngol Head Neck Surg.

[8] Smith KL, Hughes R, Myrex P (2023). Tonsillitis and Tonsilloliths: diagnosis and management. Am Fam Physician.

[9] Guangyao N, Zhengquan H, Guang L (2021). Clinical efficacy and changes of T lymphocyte subsets in children with chronic tonsillitis treated by different surgical methods. J Chin Clin Med.

[10] Yapeng Z (2020). Quality of life and analysis of influencing factors in patients with chronic tonsillitis after tonsillectomy. Sichuan J Anat.

[11] Varni JW, Burwinkle TM, Katz ER, Meeske K, Dickinson P (2002). The PedsQL in pediatric cancer: reliability and validity of the Pediatric Quality of Life Inventory Generic Core Scales, multidimensional fatigue scale, and Cancer Module. Cancer.

[12] Zhang Z, Dong M, Han Y, Lin H, Li A, Wang N, Zhang X. Application Effect of Medical Care Integration Combined with Family Intervention under the Evidence-Based Nursing Mode on Child Patients with Severe Hand-Foot-Mouth Disease and Its Influence on Intestinal Function. *Evid Based Complement Alternat Med* 2021, 2021:9599711. 10.1155/2021/9599711 PMC8523275 34671413

[13] Yiyun L, Qi T, Yuantao H (2008). Reliability and validity of the chinese version of the Children’s quality of life scale PedsQL4.0. J Sun Yat-sen Univ.

[14] Smith S (2016). Tonsillotomy: an alternative surgical option to total tonsillectomy in children with obstructive sleep apnoea. Aust Fam Physician.

[15] Mitchell RB, Archer SM, Ishman SL, Rosenfeld RM, Coles S, Finestone SA, Friedman NR, Giordano T, Hildrew DM, Kim TW (2019). Clinical practice Guideline: Tonsillectomy in Children (Update). Otolaryngol Head Neck Surg.

[16] Kubba H, Downie LS (2021). Trends in tonsillectomy surgery in children in Scotland 2000–2018. Clin Otolaryngol.

[17] Stuck BA, Genzwurker HV (2008). [Tonsillectomy in children: preoperative evaluation of risk factors]. Anaesthesist.

[18] Poddighe D, Brambilla I, Licari A, Marseglia GL (2019). Ibuprofen for Pain Control in Children: New Value for an old molecule. Pediatr Emerg Care.

[19] Varni JW, Seid M, Kurtin PS (2001). PedsQL 4.0: reliability and validity of the Pediatric Quality of Life Inventory version 4.0 generic core scales in healthy and patient populations. Med Care.

[20] Varni JW, Burwinkle TM, Seid M, Skarr D (2003). The PedsQL 4.0 as a pediatric population health measure: feasibility, reliability, and validity. Ambul Pediatr.

[21] Wang P, He Y, Zhu L (2022). Evaluation of postoperative effect and quality of life of fully expanded auricle reconstruction in children with microtia. Chin J Aesthetic Plast Surg.

[22] Liu Z, Sang L, Li W (2022). Effect of continuous nursing on postoperative quality of life and activities of daily living in children with drug-resistant epilepsy. Nurs Res.

[23] Goldstein NA, Stewart MG, Witsell DL, Hannley MT, Weaver EM, Yueh B, Smith TL, Orvidas LJ, Investigators TTS (2008). Quality of life after tonsillectomy in children with recurrent tonsillitis. Otolaryngol Head Neck Surg.

[24] Darrow DH (2008). The quality of life after tonsillectomy in children with recurrent tonsillitis. Otolaryngol Head Neck Surg.

[25] Edmonson MB, Zhao Q, Francis DO, Kelly MM, Sklansky DJ, Shadman KA, Coller RJ (2022). Association of patient characteristics with postoperative mortality in children undergoing Tonsillectomy in 5 US States. JAMA.

[26] Bell E, Dodd M, Sommerfield D, Sommerfield A, von Ungern-Sternberg BS (2021). Kids voices: exploring children’s perspective of tonsillectomy surgery. Paediatr Anaesth.

[27] Randel A (2011). AAO-HNS guidelines for Tonsillectomy in Children and Adolescents. Am Fam Physician.

[28] Hongzhen L, Kewen C, Huiqing X (2009). Quality of life after tonsillectomy for children with sleep apnea disorder. Nurs Res.

[29] Kui Y, Wenqiong X, Bo X (2018). Chinese Journal of basic and clinical General surgery. Chin J basic Clin Gen Surg.

[30] Jie L, Qingyin L (2022). Research progress of health-related quality of life in children after heart transplantation. Chin J Mod Nurs.

[31] Ping R, Yuxia Z, Yi C (2018). Influencing factors of quality of life in children with true fecal incontinence after anorectal malformation. J Nurs.

[32] Ting L, Xinyu Z, Xia Z (2021). Evaluation of quality of life and analysis of influencing factors after cochlear implantation in children with hearing impairment. Ningxia Med J.

[33] Cantarella G, Viglione S, Forti S, Minetti A, Pignataro L (2012). Comparing postoperative quality of life in children after microdebrider intracapsular tonsillotomy and tonsillectomy. Auris Nasus Larynx.

[34] Karayagmurlu A, Aytac I (2020). Pre- and postoperative quality of life and emotional/behavioural problems in children with PFAPA. Int J Pediatr Otorhinolaryngol.

[35] van Brink J, Gisselsson-Solen M (2019). Quality of life in swedish children receiving grommets - an analysis of pre- and postoperative results based on a national quality register. Int J Pediatr Otorhinolaryngol.

[36] Ma SG, Deng X, Xing L, Huang Y (2021). Postoperative health-related quality of life of patients with gynecological malignancy: a meta-analysis. Support Care Cancer.

[37] Sterian AG, Ulici A (2020). Quality of life after flatfoot surgery in the Pediatric Population. J Med Life.

[38] Sun KP, Xie WP, Liu JF, Chen Q, Cao H (2021). Quality of life analysis of children with patent ductus arteriosus after closure treatment: a single-centre study. J Paediatr Child Health.

[39] Wenli Z, Wenli Y, Jie Y (2019). Analysis of quality of life and its influencing factors in children after hematopoietic stem cell transplantation. China Compr Clin.

[40] Dorkham MC, Chalkiadis GA, von Ungern Sternberg BS, Davidson AJ (2014). Effective postoperative pain management in children after ambulatory surgery, with a focus on tonsillectomy: barriers and possible solutions. Paediatr Anaesth.

[41] Lei Z, Lijuan F (2010). Influencing factors of quality of life in 4-year-old children with congenital heart disease. Chin J Mod Nurs.

[42] Li X, Gao Y, Li Y (2021). The health-related quality of life of children after congenital blepharoptosis. J Ophthalmol.

[43] Yingling Y, Ying Y, Fanghua L (2020). Effect of continuous nursing on visual function recovery and quality of life after strabismus surgery in children. Int J Nurs.

[44] Leu GR, Links AR, Ryan MA, Walsh JM, Tunkel DE, Beach MC, Boss EF (2021). Assessment of parental choice predisposition for Tonsillectomy in Children. JAMA Otolaryngol Head Neck Surg.

[45] Huang GJ, Yang XS, Lu BQ (2022). Patient characteristics and postoperative mortality in children undergoing Tonsillectomy. JAMA.

[46] Crandall M, Lammers C, Senders C, Braun JV (2009). Children’s tonsillectomy experiences: influencing factors. J Child Health Care.

