# Supplementary material for: Quality of life in children undergoing tonsillectomy: a cross-sectional survey
Source: Ital J Pediatr. 2023 May 4;49:52. doi: 10.1186/s13052-023-01449-0 (PMC10161552; doi:10.1186/s13052-023-01449-0)
Supplement: Supplementary file 2 — Supplementary Material 2 [file 13052_2023_1449_MOESM2_ESM.docx]

Dear editor:

Manuscript ITJP-D-23-00014 entitled “Quality of life in children undergoing tonsillectomy: a cross-sectional survey", which we submitted to Italian Journal of Pediatric, has now been revised and resubmitted, the responses to the comments of the reviewers are organized one by one at the bottom of this letter.

We sincerely thank you for your tireless patience and reviewer’s wise comments on our manuscript, we marked our responses in red color to distinct from the reviewers’ suggestion, and marked the revised parts in the manuscript with red color, sincerely hope this will simplify your work.

If you have any questions, please do not hesitate to contact us.

Best regards,

Peng

Reviewer reports:

Reviewer #1: Dear Author,

The work and the analysis of the data are well done. However, I did find some minor grammatical errors (e.g. line 152: better to insert requires rather than is need). One aspect I would like to see emphasised is the role of the nurse in the management of children in the post-operative period: for example, you could emphasise the role in providing the correct instructions to follow at home. It would also be appropriate to suggest how to improve the perioperative or outpatient care of these patients from a nursing perspective.

Thank you for your kind suggestions, we have carefully checked and revised the language accordingly to make it more approperiate and fluent. Besides, we have added more explaination and discussions on the the role of the nurse in the management of children in the post-operative period as you kindly suggested, please see the revised manuscript.

Reviewer #2: Well written article and good data analysis, adequate sample size. Some language revisions required

Thank you for your kind suggestion, we have carefully checked and corrected the grammar as possible as we can, see the revised manuscript. Besides, since we are not native English speaker, we have sent our manuscript to for native language editing, see the revised manuscript and enclosed language editing certificate.

Reviewer #3: INTRODUCTION

- "Therefore, the timely and effective treatment and nursing care are essential to the prognosis of children with tonsillitis." I would like to ask the authors what they mean for "nursing care" in this clinical setting and to support this statement with references, if it is not removed.

Thank you for your discreet consideraiton, we have removed the “nursing care” and added reference(Ref 8) to support this statement as you kindly suggested, please see the revised introduction section.

- I think the authors should define "chronic tonsillitis".

Thank you, we have defined and explained the "chronic tonsillitis" as you kindly suggested, please see the revised introduction section.

- "Tonsillectomy is a common treatment for chronic tonsillitis at present, and the quality of life of postoperative patients is one of the key points of clinical concern[5]." Ref. 5 is from a local journal and does not seem to be appropriate for supporting this central concept according to the aims of the study.

Thank you for your kind suggesion, we have checked and updated the references(Ref 9, 10) as you kindly suggested, please see the revised introduction section.

- "In recent years, the incidence of tonsillitis and enlarged tonsil is increasing with the aggravation of environmental pollution[1]." More concepts about the predisposing factors to tonsillitis and, in general, recurrent respiratory infections in children should be provided. For instance, the role of passive smoking should be also considered and mentioned (as discussed in: J Interferon Cytokine Res. 2009 Aug;29(8):427-32. doi: 10.1089/jir.2008.0108); also the role of infectious agents should be mentioned (see: BMC Pediatr. 2022 Sep 19;22(1):553. doi: 10.1186/s12887-022-03613-7.)

Thank you for your kind suggestions, we have cited this reports(Ref 2, 3) for introduction as you kindly suggested, please see the revised introduction.

METHODS

- study design should be declared at the beginning.

Thank you, we have explained the study design as the begining of method section as you kinldy suggested, please see the revised manuscript.

- the sentence related to the informed consent should be revised and clarified.

Thank you, we have explained the informed consent as you kindly suggested, please see the revised method section.

- IRB approval date should be also reported.

Thank you, we have explained the IRB approval date as you kindly suggested.

- "All children were given thrombin and dexamethasone 2 days after operation, budesonide suspension was atomized, cold liquid food was ordered 6 hours after operation, semi-fluid diet was allowed on the 2nd day, the temperature should not be too high, and soft food was taken from the3rd day to the period from the 3rd day to the complete exfoliation of the albino". Can the authors also clarify the use of analgesic therapy, including ibuprofen or NSAIDs (refer to: Pediatr Emerg Care. 2019 Jun;35(6):448-453. doi: 10.1097/PEC.0000000000001505.).

Thank you for your kind sugeestions, we have added more explaination as you kindly suggested, please see the revised manuscript.

- Is it possible to provide the questionnaire as a supplementary material. That would allow a better assessment of the results by the readership as well.

Thank you for your kind suggestion, we can provde the questionnaire on request whenver necessary.

RESULTS

- Overall, I suggest expanding this section by providing a detailed description of the main and more significant findings.

Thank you for your kind suggestions, we have explained the findings as you kindly suggested, please see the revised results section.

- Moreover, this section could be organized in subsections with specific subheading, after improving the results description, including numerical values and level of statistical significance.

Thank you for your kind suggestion, we have added more subsections and explaination on our findings to make more logic and specific, please see the the revised results section.

DISCUSSION

- The authors should start the discussion by listing and highlighting the main findings and novelty of the present study. Then, each point should be individually discussed in light of the previous literature. Notably, there are some previous studies addressing specifically the issue of quality of life after tonsillectomy in children with recurrent tonsillitis, which have not been considered by the authors (e.g. Otolaryngol Head Neck Surg. 2008 Jan;138(1 Suppl):S9-S16. doi: 10.1016/j.otohns.2006.12.029; Otolaryngol Head Neck Surg. 2008 Oct;139(4):608; author reply 608-9. doi: 10.1016/j.otohns.2008.07.013; others). The authors could use them to compare the differences compared to previous periods and also to compare the different methodological approach to assess this aspect.

Thank you for your kind suggestions, we have explained more about our findings and compared to those reports to provide more implication for clinical tonsillitis treatment, please see the revised discussion section.

- A mention regarding different post-operative approaches, including the use of different NSAIDs (see previous comments and related references), should be made in the discussion.

Thank you for your kind suggestion, we inclded the different post-operative approaches for discussion as you kindly suggested, please see the revised discussion section.

- The discussion on limitations should be expanded. I also noticed that there are no mention regarding exposure to passive smoke, other environmental factors, or allergy.

Thank you for your kind suggestion, we have added those limitiations for consideration as you kindly suggested, please see the revised manuscript.

REFERENCES

- to be updated and completed based on the improvement of the revised discussion and according to the previous suggestions.

Thank you, we have updated the references as you kindly suggested, please see the revised manuscript. We really appreicate your kind help in improving our manuscript, thank you.
